# Supplementary material for: Content-rich biological network constructed by mining PubMed abstracts
Source: BMC Bioinformatics. 2004 Oct 8;5:147. doi: 10.1186/1471-2105-5-147 (PMC528731; doi:10.1186/1471-2105-5-147)
Supplement: Additional File 2 — The original results of the above study (non-essential files are deleted to keep the file size under the limit set by BMC bioinformatics). [file 1471-2105-5-147-S2.bz2 › chilibotAdditionalFile2/dip10/85ID8702708E312/html/TRAF1_TRAF2.html]

 


 **TRAF1** and **TRAF2** 
  
Found 193 abstracts in PubMed, retrieved 10.  
 

 What does Google say? 
 PDF only 
| .edu only 

---

**Interactive relationship** (e.g. stimulation, inhibition, etc)

**Neutral relationship**- Here, we show that  **TRAF1**  differentially affects  **TRAF2**  recruitment and activation of NF kappaB by members of the TNF receptor family.  Ref: 12709429 J Biol Chem< MedlineTA>J Biol Chem,

**Non-interactive relationship** (e.g. studied together, co-existance, homology, etc.)

- We carried out comparative in situ hybridization analysis of five Traf  [ **TRAF1** ]  genes  **Traf1** ,  **Traf2** , Traf3, Traf4 and Traf6 during murine odontogenesis from the formation of the epithelial thickening to the early bell stage.  Ref: 12711536 Gene Expr Patterns, 2003
- Expression of  **Traf1** ,  **Traf2**  and Traf6 were observed in the outer edges of the bud epithelium whereas Traf3 was strongly expressed at the tip of the bud epithelium.  Ref: 12711536 Gene Expr Patterns, 2003
- Our results suggest that  **TRAF1**  and  **TRAF1**  164 416 exert their regulatory effects on receptor induced NF kappaB activation not only by modulation of  **TRAF2**  receptor interaction but especially  **TRAF1**  164 416 also by directly targeting the IKK complex.  Ref: 12709429 J Biol Chem< MedlineTA>J Biol Chem, 2003
- Western blot analysis further demonstrated that OIP 1 significantly decreased TNF receptor associated factor 2  [ **TRAF2** ]  TRAF  [ **TRAF1** ]  2 expression in RAW 264.7 cells.  Ref: 12619930 J Bone Miner Res, 2003
- However, NFkappaB stimulation by AII differs from that of TNF alpha since a TNF receptor associated factor 2  [ **TRAF2** ]  TRAF  [ **TRAF1** ]  2 dominant negative mutant does not prevent AII mediated NFkappaB activation.  Ref: 12704649 J Cell Physiol, 2003
- We have previously reported that the HCV NS5A protein interacts with TRAF  [ **TRAF1** ]  domain of TNF receptor associated factor 2  [ **TRAF2** ]   **TRAF2** .  Ref: 12796506 J Biol Chem< MedlineTA>J Biol Chem, 2003
- **Traf2**  showed restricted expression in the internal enamel epithelium of the bell stage while expression of  **Traf1** , Traf3, Traf4 and Traf6 were observed in both the internal and the external enamel epithelium.  Ref: 12711536 Gene Expr Patterns, 2003
- The constitutive expression of mRNA for TNF receptors TNFR, including TNFRI and TNFRII, and the adapter molecules, such as the TNF receptor associated death domain protein TRADD, Fas associated death domain protein FADD, receptor interacting protein RIP and TNF receptor associated factor 2  [ **TRAF2** ]  TRAF  [ **TRAF1** ]  2 were analyzed by reverse transcriptase RT PCR in bone marrow samples from control, MDS and AML cases.  Ref: 12681357 Leuk Res, 2003
